# Supplementary material for: Ankle muscles drive mediolateral center of pressure control to ensure stable steady state gait
Source: Sci Rep. 2021 Nov 2;11:21481. doi: 10.1038/s41598-021-00463-8 (PMC8563802; doi:10.1038/s41598-021-00463-8)
Supplement: Supplementary file 1 — Supplementary Information. [file 41598_2021_463_MOESM1_ESM.pdf]

Supplementary material

S1

We instructed participants to keep placing their feet in a similar orientation (straight ahead) throughout all conditions, to avoid a toeing out strategy, especially in the ankle moment constrained condition, where we wanted to limit the CoP shift during single stance. Following Fig S1, showing the toe-out angle of the 30 included participants, it appears most participants followed these instructions. The toe-out angle (with respect to the global vertical axis) showed to be similar during steady-state and ankle moment constrained walking, given moderate evidence against any difference at both normal (BF_{10} =0.319) and slow (BF_{10} = 0.196) speed.

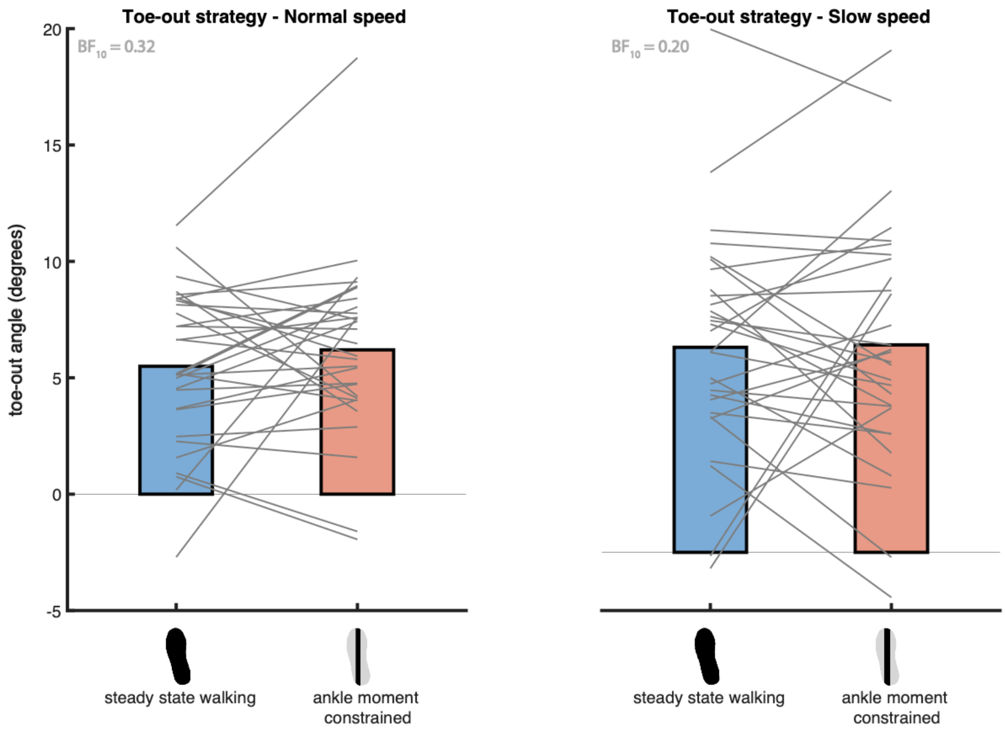

S1 Fig 1. The toe-out angle during steady-state and ankle moment constrained walking. The angles are expressed with respect to the feet pointing straight ahead (0 degrees). Positive and negative angles correspond to respectively toeing-out and toeing-in. The grey lines connect individual data points. The Bayes factors (BF_{10}) represent the degree of evidence against an increased toe-out angle in the ankle moment constrained condition, as compared to steady-state walking. This figure was created using Matlab 2021a (https://www.mathworks.com/products/matlab.html) and Adobe Illustrator CC 2018 (https://www.adobe.com/nl/products/illustrator.html).

S2

Only including medial steps for the ankle strategy model

One could argue, that stepping too lateral does not threaten stability like stepping too medial does. Stepping too medial can lead to a sideward fall, whereas a "fall" induced by stepping too lateral would merely accelerate the body towards the subsequent stance foot. Therefore, we also considered the ankle strategy model while only including steps which were too medial.

The negative relationship between foot placement error and the CoP shift was retained in the model including only medial steps. When testing the regression coefficients against zero using a Bayesian one-sample t-test, extreme evidence was found for the ankle strategy models at both speeds.

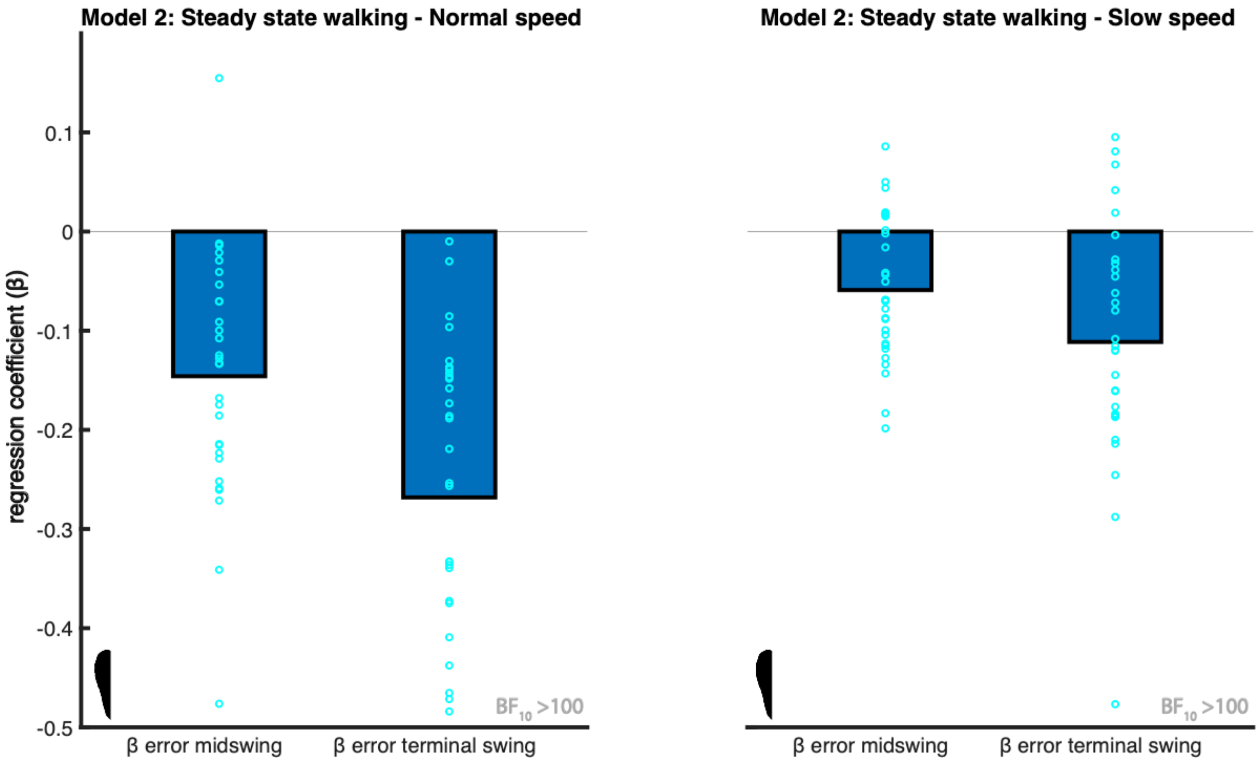

S2 Fig 1. Mean regression coefficients of the ankle strategy model (2). The predictors (β) are foot placement error at mid-swing and terminal swing. In contrast to Fig 3, here only the foot placement errors corresponding to the too medial steps were included, as indicated by half a steady-state walking condition foot print. The light blue circles represent individual data points. The negative relationship shows ankle moments accommodate for stepping inaccuracies, by shifting the CoP more lateral when stepping more medial. The Bayes factors (BF_{10}) denote the degree of evidence supporting the regression coefficients to be different from zero. This figure was created using Matlab 2021a (https://www.mathworks.com/products/matlab.html) and Adobe Illustrator CC 2018 (https://www.adobe.com/nl/products/illustrator.html).

S2 Fig 2a shows that the relative explained variance appears to be higher when including all steps in the model as compared to including only too medial steps, with foot placement error at mid-swing as predictor. Based on the mid-swing foot placement error this was supported by very strong evidence at normal walking speed (BF_{10} = 79.173). Yet, at a slow walking speed there is anecdotal evidence against this observation (BF_{10} = 0.683). Similarly, S2 Fig 2b shows a trend for a higher relative explained variance when all steps are included in the model with foot placement error at terminal swing as predictor. Testing this observation (two-tailed), we

found extreme evidence supporting this observation at normal speed (BF_{10} = 990100.061) and anecdotal evidence against this observation at slow speed (BF_{10} = 0.476).

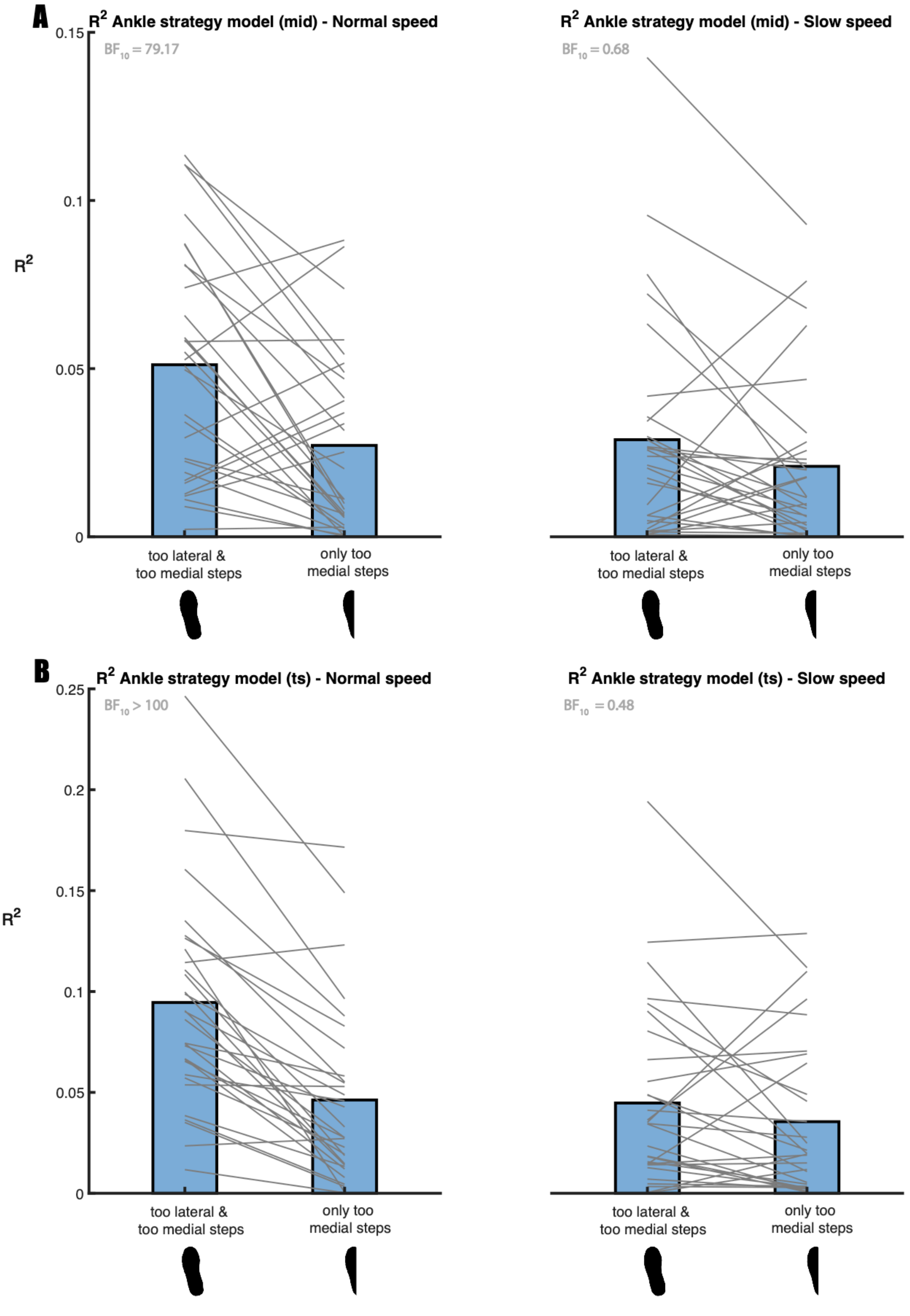

S2 Fig 2. The relative explained variance (R^2) of the ankle strategy model (2). R^2 was computed for the model with the foot placement error at mid-swing (panel A) and terminal swing (panel B). A comparison is made between including all (full steady-state walking condition foot print) as compared to only too medial steps (half a steady-state walking condition foot print), with the foot placement error at mid-swing as predictor. For most participants, the ankle strategy model, explained a higher percentage of the variance in CoP shifts, when both the too lateral and the too medial steps are included. Grey lines connect individual data points. The Bayes

factors (BF_{10}) denote the degree of evidence. This figure was created using Matlab 2021a (https://www.mathworks.com/products/matlab.html) and Adobe Illustrator CC 2018 (https://www.adobe.com/nl/products/illustrator.html).

S3

Relative explained variance ankle strategy muscle model (3)

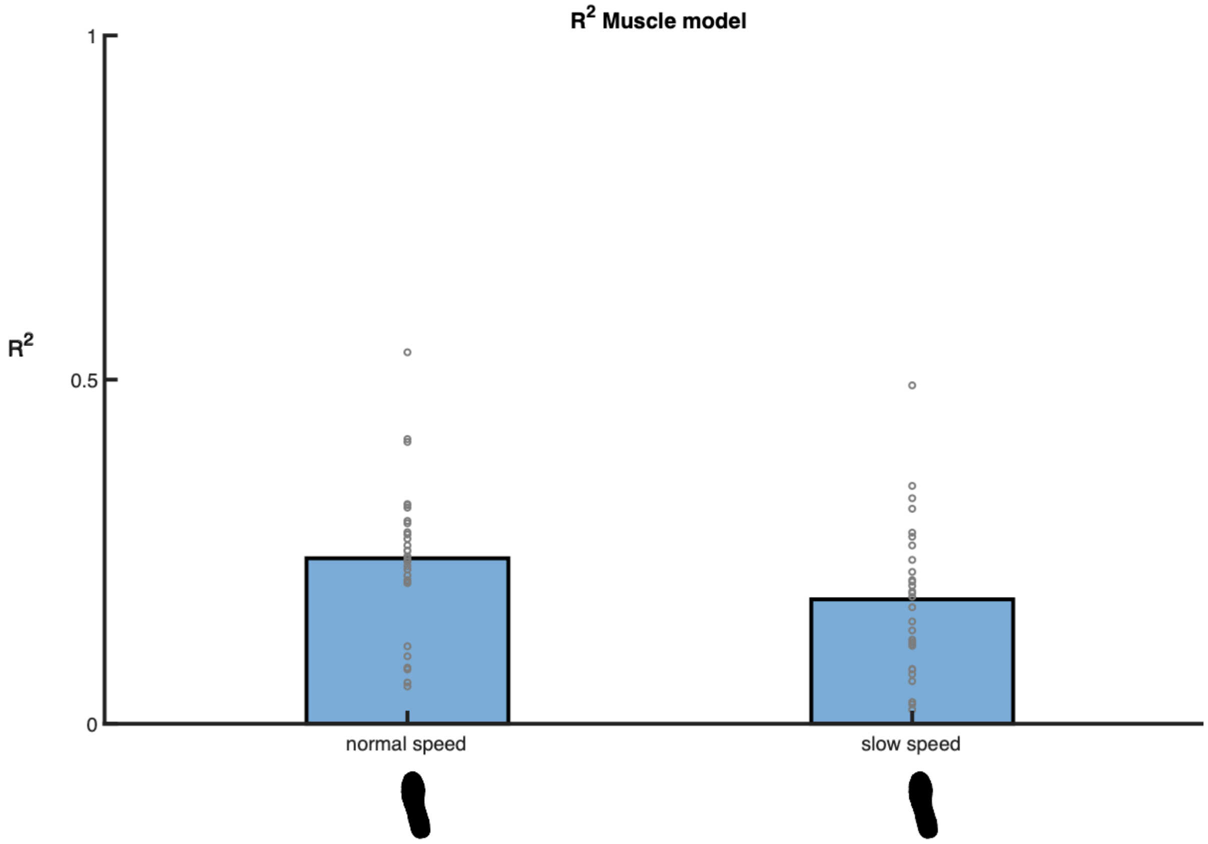

S3 Fig 1. **R^2 of the ankle strategy muscle model (3)**. The bars represent the average R^2 at respectively normal (left) and slow (right) walking speed**.** Grey circles represent individual data points. This figure was created using Matlab 2021a (https://www.mathworks.com/products/matlab.html) and Adobe Illustrator CC 2018 (https://www.adobe.com/nl/products/illustrator.html).
